# Metastatic Rhabdomyosarcoma: Results of the European *Paediatric* Soft Tissue Sarcoma Study Group MTS 2008 Study and Pooled Analysis With the Concurrent BERNIE Study

**DOI:** 10.1200/JCO.21.02981

**Published:** 2022-06-16

**Authors:** Reineke A. Schoot, Julia C. Chisholm, Michela Casanova, Veronique Minard-Colin, Birgit Geoerger, Alison L. Cameron, Beatrice Coppadoro, Ilaria Zanetti, Daniel Orbach, Anna Kelsey, Timothy Rogers, Cecile Guizani, Markus Elze, Myriam Ben-Arush, Kieran McHugh, Rick R. van Rijn, Sima Ferman, Soledad Gallego, Andrea Ferrari, Meriel Jenney, Gianni Bisogno, Johannes H.M. Merks

**Affiliations:** ^1^ Princess Máxima Centre for Paediatric Oncology, Utrecht, the Netherlands; ^2^ Children and Young Peoples Unit, Royal Marsden Hospital and Institute of Cancer Research, Sutton, Surrey, United Kingdom; ^3^ Paediatric Oncology Unit, Fondazione IRCCS Istituto Nazionale dei Tumori, Milan, Italy; ^4^ Gustave-Roussy Cancer Campus, Department of Paediatric and Adolescent Oncology, Université Paris-Saclay, Villejuif, France; ^5^ Gustave-Roussy Cancer Campus, INSERM U1015, Université Paris Saclay, Villejuif, France; ^6^ Bristol Haematology and Oncology Centre, University Hospitals Bristol and Weston NHS Foundation Trust, Bristol, United Kingdom; ^7^ Haematology Oncology Division, Department of Women's and Children's Health, University of Padova, Padova, Italy; ^8^ SIREDO Oncology Center, Institut Curie, PSL University, Paris, France; ^9^ Department of Paediatric Histopathology, Royal Manchester Children's Hospital, Manchester, United Kingdom; ^10^ Department of Pediatric Surgery, University Hospitals Bristol and Weston NHS Foundation Trust, Bristol, United Kingdom; ^11^ F. Hoffmann-La Roche Ltd, Basel, Switzerland; ^12^ Joan and Sanford Weill Pediatric Hematology Oncology and Bone Marrow Transplantation Division, Ruth Rappaport Children's Hospital, Rambam Medical Center, Haifa, Israel; ^13^ Department of Radiology, Great Ormond Street Hospital for Children NHS Foundation Trust, London, United Kingdom; ^14^ Department of Radiology and Nuclear Medicine, Emma Children's Hospital, Amsterdam UMC, University of Amsterdam, the Netherlands; ^15^ Instituto Nacional de Câncer, Pediatric Oncology Department, Rio de Janeiro, RJ, Brazil; ^16^ Pediatric Oncology, Vall d'Hebron University Hospital, Barcelona, Spain; ^17^ Department of Paediatric Oncology, Children's Hospital for Wales, Heath Park, Cardiff, United Kingdom

## Abstract

Outcome for patients with metastatic rhabdomyosarcoma (RMS) is poor. This study presents the results of the MTS 2008 study with a pooled analysis including patients from the concurrent BERNIE study.

**PATIENTS AND METHODS:**

In MTS 2008, patients with metastatic RMS received four cycles of ifosfamide, vincristine, and actinomycin D (IVA) plus doxorubicin, five cycles of IVA, and 12 cycles of maintenance chemotherapy (low-dose cyclophosphamide and vinorelbine). The BERNIE study randomly assigned patients to the addition or not of bevacizumab to the same chemotherapy. Local therapy (surgery/radiotherapy) was given to the primary tumor and all metastatic sites when feasible.

**RESULTS:**

MTS 2008 included 270 patients (median age, 9.6 years; range, 0.07-20.8 years). With a median follow-up of 50.3 months, 3-year event-free survival (EFS) and overall survival (OS) were 34.9% (95% CI, 29.1 to 40.8) and 47.9% (95% CI, 41.6 to 53.9), respectively. In pooled analyses on 372 patients with a median follow-up of 55.2 months, 3-year EFS and OS were 35.5% (95% CI, 30.4 to 40.6) and 49.3% (95% CI, 43.9 to 54.5), respectively. Patients with ≤ 2 Oberlin risk factors (ORFs) had better outcome than those with ≥ 3 ORFs: 3-year EFS was 46.1% versus 12.5% (*P* < .0001) and 3-year OS 60.0% versus 26.0% (*P* < .0001). Induction chemotherapy and maintenance appeared tolerable; however, about two third of patients needed dose adjustments during maintenance.

**CONCLUSION:**

Outcome remains poor for patients with metastatic RMS and multiple ORFs. Because of the design of the studies, it was not possible to determine whether the intensive induction regimen and/or the addition of maintenance treatment resulted in apparent improvement of outcome compared with historical cohorts. Further studies, with novel treatment approaches are urgently needed, to improve outcome for the group of patients with adverse prognostic factors.

## INTRODUCTION

Rhabdomyosarcoma (RMS) is a very aggressive tumor with a strong tendency to metastasize. Outcome in patients with localized disease is generally good,^1,2^ but outcome for patients with metastatic RMS remains poor with 3-year overall survival (OS) of 34%-56%.^3,4^ Various attempts to increase treatment intensity failed to improve survival (eg, high-dose chemotherapy with stem-cell support)^5-8^ or resulted in very limited improvement in selected subgroups of patients (dose-compressed chemotherapy).^4^

**CONTEXT**

**Key Objective**
To evaluate the efficacy of the addition of doxorubicin to standard chemotherapy (ifosfamide, vincristine, and actinomycin D) and the introduction of 1-year maintenance chemotherapy (cyclophosphamide and vinorelbine) in patients with metastatic rhabdomyosarcoma (RMS).
**Knowledge Generated**
Outcomes in this study seem improved compared with historical cohorts, but owing to the design of the study, it remains unclear whether this is attributable to the addition of doxorubicin or maintenance chemotherapy, or may be explained by more consistent application of local therapy. Outcome for metastatic patients with adverse prognostic factors remains poor.
**Relevance**
The ifosfamide, vincristine, and actinomycin D with the addition of doxorubicin regimen followed by 1 year of maintenance chemotherapy is the current standard for patients with metastatic RMS in Europe, but further studies are needed to validate the role of doxorubicin and role and duration of maintenance chemotherapy. Introduction of new strategies in frontline treatment is needed, to reduce treatment failure in patients with metastatic RMS.

The European *pediatric* Soft tissue sarcoma Study Group (E*p*SSG) has collaborated in three studies in newly diagnosed RMS in recent years. The E*p*SSG RMS 2005 study (conducted from 2005 to 2016) explored the added value of dose intense doxorubicin in combination with standard ifosfamide, vincristine, and actinomycin-D (IVADo) chemotherapy, and the role of 6 months of maintenance chemotherapy following completion of standard therapy in high-risk localized disease.^1,2^ Concurrently with the opening of RMS 2005, the E*p*SSG and Innovative Therapies for Children with Cancer (ITCC) collaborated with Roche in the BERNIE study, a pharma-sponsored study for patients with metastatic soft tissue sarcoma.^9^ In this open-label, randomized phase II study (conducted from 2008 to 2013), patients received standard induction chemotherapy followed by a year of maintenance treatment with vinorelbine and low-dose cyclophosphamide. Patients were randomly assigned to receive or not receive bevacizumab. The BERNIE study recruited 152 patients, including 102 with RMS. No benefit of bevacizumab on event-free survival (EFS) was demonstrated.^9^

Since the BERNIE study was open in a limited number of sites and had stringent inclusion/exclusion criteria, the single-arm E*p*SSG MTS 2008 study was introduced as an amendment to RMS 2005, using the same induction and maintenance chemotherapy as the BERNIE study but without bevacizumab, to capture data on patients with metastatic RMS who did not enter the BERNIE study.

The current study reports treatment, toxicity, and outcome of patients with metastatic RMS treated within the MTS 2008 study. As a secondary objective, to address potential selection bias introduced by the concurrent BERNIE study, we performed a pooled analysis of MTS 2008 and BERNIE study results. For the purpose of the current analysis, the results from the BERNIE study were updated and mature OS data were reported for the first time.

## PATIENTS AND METHODS

### Study Design and Participants

MTS 2008 was an academic, international, prospective study (ClinicalTrials.gov identifier: NCT00379457) involving 74 hospitals across 11 countries. Patients < 21 years old with a histologic diagnosis of RMS (excluding pleomorphic RMS) with distant metastatic disease, < 8 weeks between diagnostic surgery/biopsy and start of chemotherapy, and who had received no prior chemotherapy or radiotherapy were eligible.

Concurrent with the MTS 2008 study, patients were recruited to the BERNIE study (BO20924/ITCC-006; ClinicalTrials.gov identifier: NCT00643565).^10^ Updated data from the final BERNIE Clinical Study Report were used for the current OS analyses.

Detailed eligibility criteria for both studies can be found in the Data Supplement (online only).

Both studies were conducted in accordance with the Declaration of Helsinki and the Good Clinical Practice guidelines. All participating centers were required to obtain approval from their local authorities and ethics committees, and written informed consent from patients and/or their parents or legal guardians.

### MTS 2008 Treatment

Induction chemotherapy comprised 9 × 3-weekly cycles including four cycles of IVADo and five cycles of IVA (Data Supplement).^1^ Maintenance chemotherapy comprised 12 × 28-day cycles of intravenous vinorelbine and low-dose oral cyclophosphamide (Data Supplement).^2^ Chemotherapy was identical to the standard treatment arm of the BERNIE study, wherein the investigational arm patients received the same chemotherapy treatment with the addition of bevacizumab every 3 weeks on day 1 of each cycle and every 2 weeks during maintenance.^9^ Growth factors were allowed at the physicians' discretion. Adverse events (AEs) were graded according to the Common Terminology Criteria for Adverse Events (version 3.0). Only the following AEs ≥ grade 3 were recorded: infection (proven or suspected), cardiomyopathy, neuropathy, mucositis, or veno-occlusive disease.

Surgical resection of residual primary tumor was considered after the sixth chemotherapy course (week 19 onward), generally avoiding mutilating surgery. Resections were only recommended if a R0 (microscopically margin-negative resection) or R1 (macroscopic resection with positive microscopic margins) resection seemed feasible. R2 resection (macroscopic residual) and radical lymph node dissections were not recommended.

Radiotherapy was recommended to the primary tumor site and, if feasible, to all metastatic sites, regardless of response to chemotherapy, starting concomitantly with the seventh chemotherapy cycle. Dose to the primary tumor was adapted to primary tumor response and histology (Data Supplement). Whole lung radiotherapy was recommended for patients with one or more lung metastases. Since the number of metastatic sites and the size of the metastases can vary and can be very extensive, the local multidisciplinary teams considered each patient individually, involving the study's radiotherapy coordinator if needed.

### Response Assessment

First response assessment was scheduled after three cycles of chemotherapy (week 9). In case of insufficient response (≤ 1/3 volume reduction), patients were eligible for second-line treatment. Alternatively, participation in the VIT-0910 study was considered, evaluating the addition of temozolomide to the combination of vincristine and irinotecan.^10^ A second response assessment was scheduled preceding local treatment, after six cycles of chemotherapy (week 18). Response of the primary tumor was measured as volume reduction; response of metastatic lesions was measured according to the RECIST version 1.0.^11,12^

### Statistical Methods

Differences between cohorts were compared with the chi square or Fisher's exact test, depending on frequency distribution of each variable. Survival probabilities were estimated by use of the Kaplan-Meier method and the log-rank test. EFS was defined as the time between diagnosis and disease progression, recurrence, refusal of therapy, suspension of treatment due to toxicity, or death due to any cause. OS was defined as the time from date of diagnosis up to death for any reason. Patients still alive at the end of the study or lost to follow-up were censored, both in the EFS and OS analyses, at the date of last observation. EFS and OS were evaluated by prognostic factors identified in the joint European-Children's Oncology Group study published by Oberlin et al^3^ (Oberlin risk factors [ORFs]), being age, site, bone or bone marrow involvement, and number of metastatic sites. Of note, parameningeal primary tumor site was grouped as favorable by Oberlin et al.^3^

## RESULTS

### Patient Characteristics MTS 2008

Between October 2010 and December 2016, 324 patients were registered in the MTS 2008 study; 54 patients were reported not eligible for the following reasons: age > 21 years (n = 11), included in another protocol (n = 6), other previous treatments (n = 16), no written informed consent (n = 7), pathology not available for central review (n = 5), interval from surgery to chemotherapy start exceeded 8 weeks (n = 7), staging error (n = 1), and RMS diagnosis not confirmed by pathology (n = 1; Data Supplement). The remaining 270 patients (median age, 9.6 years; range, 0.1-20.8 years; unfavorable histology, 57%) were included in the MTS 2008 analysis (Table 1).

**TABLE 1.**
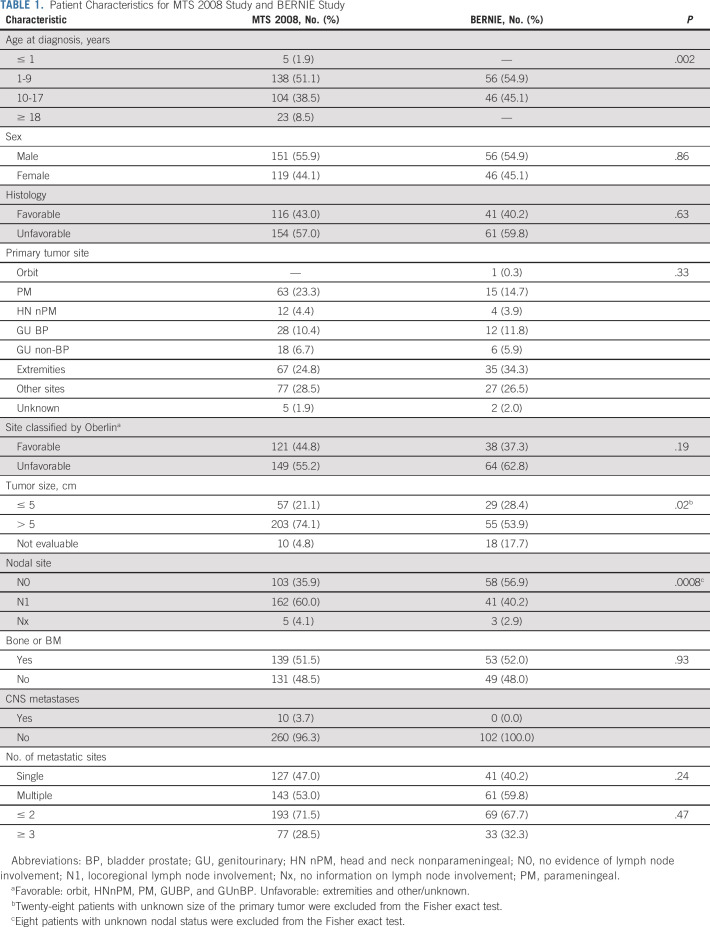
Patient Characteristics for MTS 2008 Study and BERNIE Study

### Treatment Characteristics

Standard induction chemotherapy was completed as scheduled in 218/259 patients (missing data, n = 11); 12 were switched to second line treatment for stable disease (n = 11) or serious adverse event (n = 1), and in 29 patients, induction chemotherapy was discontinued because of progressive disease (n = 25), death (n = 3), or treatment refusal (n = 1). Of the 218 patients completing induction treatment, 181 (83%) commenced maintenance chemotherapy and 103/181 (57%) completed all 12 cycles. Reasons for discontinuation of maintenance chemotherapy were death or disease recurrence (n = 60), toxicity (n = 4), error (n = 7), patient's choice (n = 4), change in diagnosis (n = 1), or unknown (n = 2).

Data on primary tumor response were available for 248/270 patients; the majority of patients (228/248, 92%) achieved sufficient response (≥ 33% volume reduction of the primary tumor) during induction chemotherapy, including 17 complete remissions. Response of metastatic lesions was not available for all sites, but overall, 182/560 (33%) metastatic sites were in complete remission after two-four courses of induction chemotherapy (Data Supplement).

Local treatment included delayed (ie, week 19) resection of the primary tumor in 66 patients; 40 patients had R0, 17 R1, and nine R2 resection. In 20 patients, locoregional lymph node exploration was performed at delayed surgery, by surgical biopsy (n = 13), lymphadenectomy (n = 6), or both (n = 1). Data on radiotherapy were available for 256/270 patients; radiotherapy was administered in 211 patients; 45 patients were not irradiated. Reasons for withholding radiotherapy were early disease progression (n = 14), physicians' decision (n = 13), very young age (n = 7), parental refusal (n = 3), early death (n = 3), widespread disease at diagnosis (n = 2), and reason unknown (n = 3). In total, 194 patients received radiotherapy to the primary tumor with a median dose of 50.4 Gy (range, 18-68.6 Gy) and 89 patients were irradiated at one or more metastatic sites (median dose, 30 Gy; range, 9-59.4 Gy).

### Toxicity

During induction chemotherapy, most common grade 3/4 AEs (evaluated in 218 patients) were infection (grade 3; n = 118, grade 4; n = 5), followed by mucositis (grade 3; n = 66, grade 4; n = 9) and neuropathy (grade 3; n = 22, grade 4; n = 2). Veno-occlusive disease and cardiac AEs were rare, with three and two patients developing grade ≥ 3 toxicity, respectively. During induction chemotherapy, courses were modified in about 20% of the patients (see Table 2 for details). During maintenance therapy, 22/100 (22%) patients had grade 3 infection, two (2%) grade 3 neuropathy, and one (1%) a grade 3 cardiac AE. Chemotherapy was modified according to protocol guidelines: in approximately 40% of patients during the first maintenance cycle, up to 60% during the second cycle, and remained stable around 60% thereafter (Table 2). Reasons for treatment reduction were mostly myelotoxicity or infection.

**TABLE 2.**
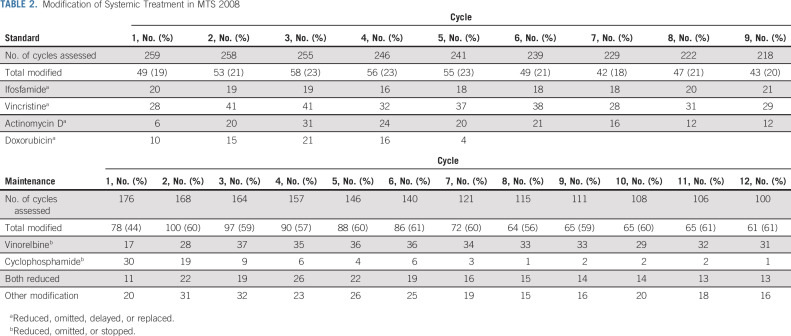
Modification of Systemic Treatment in MTS 2008

### Outcome

**FIG 1.**
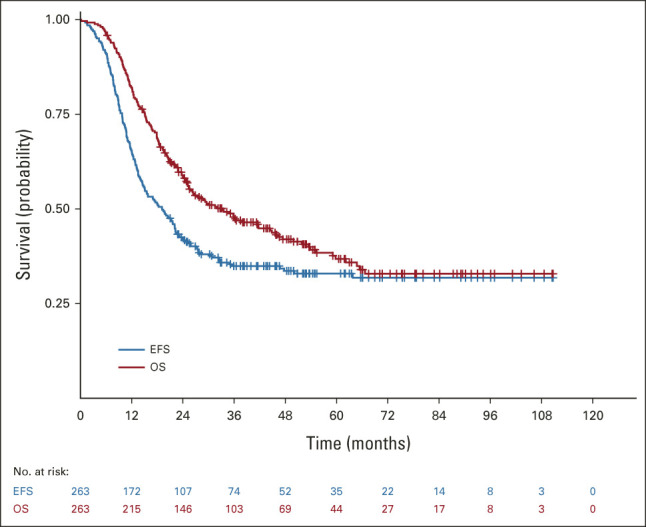
EFS and OS of patients in MTS 2008. EFS, event-free survival; OS; overall survival.

Median follow-up duration was 50.3 months (range, 6.3-110.7 months). For the 173 patients who experienced an EFS event, the median time from diagnosis was 11.6 months (range, 0.2-63.8 months). The 3-year EFS was 34.9% (95% CI, 29.1 to 40.8) and 3-year OS was 47.9% (95% CI, 41.6 to 53.9; Fig 1). Of 270 patients, 125 (46%) developed progressive disease, had insufficient response, relapsed, or died during (or at completion of) induction (n = 65) or maintenance (n = 60) treatment.

### Pooled Analysis

**FIG 2.**
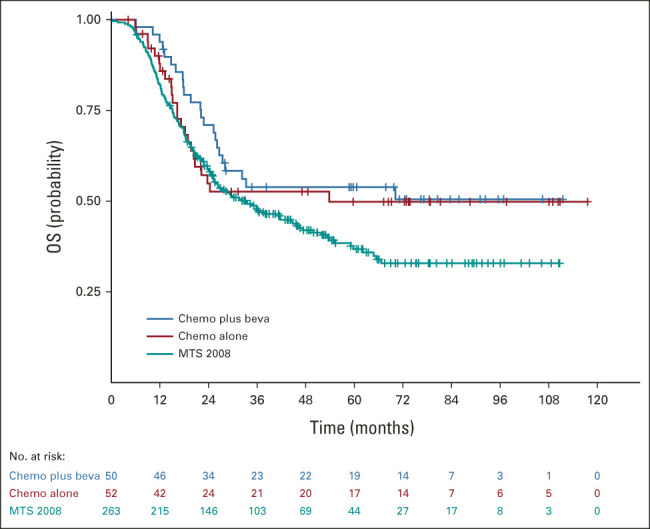
OS by treatment cohort. Beva, bevacizumab; chemo, chemotherapy; OS, overall survival.

Overall, 102 consecutive treated patients from the BERNIE cohort (50 were randomly assigned to the experimental bevacizumab arm) were analyzed. Patients < 6 months and ≥ 18 years and patients with brain metastases were ineligible for the BERNIE study, introducing a difference in age distribution and the number of patients with brain metastases between cohorts (Table 1). In addition, more patients with locoregional lymph node involvement (*P* = .0008) and a large primary tumor (> 5 cm; *P* = .02) were included in the MTS 2008 study. Median follow-up duration for patients in the BERNIE study was 71.8 months (range, 0.03-117.6 months). The 3-year EFS was 37.0% (95% CI, 26.2 to 47.8) and 3-year OS was 53.1% (95% CI, 42.4 to 62.6). OS rates for both BERNIE arms were comparable (Fig 2).

Outcome data were available for 365/372 patients (98%) in the pooled analysis. At last follow-up, 164 patients (45%) were alive. With a median follow-up of 55.2 months (range, 0.03- 117.6 months), the 3-year EFS and 3-year OS for the pooled cohort were 35.5% (95% CI, 30.4 to 40.6) and 49.3% (95% CI, 43.9 to 54.5), respectively (Table 3). The 3-year EFS was similar for patients in the MTS 2008 study and the BERNIE study (*P* = .54), and 3-year OS was lower for patients in the MTS 2008 study compared with the BERNIE study (*P* = .03; Fig 2).

**TABLE 3.**
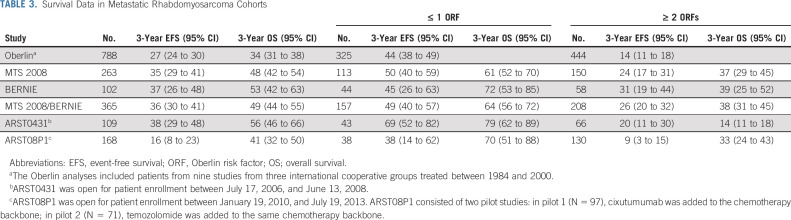
Survival Data in Metastatic Rhabdomyosarcoma Cohorts

**FIG 3.**
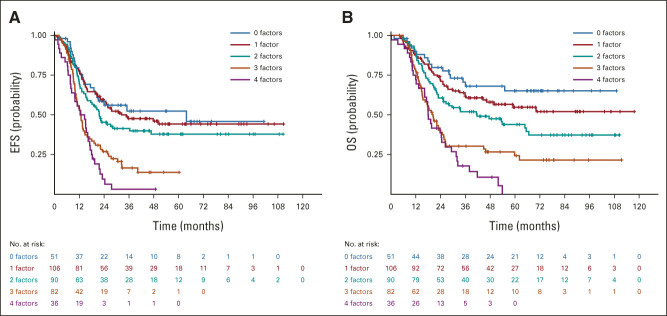
(A) EFS by ORFs for pooled MTS 2008 and BERNIE cohorts. (B) OS by ORFs for pooled MTS 2008 and BERNIE cohorts. EFS, event-free survival; ORF, Oberlin risk factor; OS, overall survival.

We performed subgroup analyses, excluding patients < 1 or ≥ 18 years old (not enrolled in the BERNIE study) or with brain metastases (exclusion criterion; Data Supplement), to adjust for the difference in patient characteristics between the MTS 2008 and BERNIE cohorts. There was no significant difference in 3-year OS between MTS 2008 (3-year OS 51.8% [95% CI, 44.9 to 58.2]) and BERNIE (3-year OS 53.1% [95% CI, 42.4 to 62.6]) for this specific patient subgroup (*P* = .14). Overall, 106/372 (28.3%) of patients were < 10 years with embryonal histology. Follow-up data were available for 103/106 patients; the 3-year EFS was 54.3% (95% CI, 43.9 to 63.3) and 3-year OS was 63.5% (95% CI, 53.2 to 72.2). EFS and OS by ORFs are shown in Figure 3. Patients who had 0-2 ORFs had a significantly better outcome than those with 3-4 ORFs: 3-year EFS 46.1% versus 12.5% (*P* < .0001) and 3-year OS 60.0 versus 26.0% (*P* < .0001, Fig 2B, Data Supplement).

## DISCUSSION

This study suggests a moderate improvement in outcome for patients with metastatic disease compared with historical cohorts, similar to the results described for the COG ARST0431 study, which used a dose-intense multiagent schedule and radiotherapy sensitization with irinotecan.^4^ Owing to the design of the studies, it was not possible to determine whether the addition of doxorubicin or the introduction of maintenance treatment contributed to the apparent improvement. Additionally, we present here the first mature OS results for patients with metastatic RMS treated in the BERNIE study,^9^ confirming that the addition of bevacizumab to the MTS 2008 backbone did not improve OS for this group of patients. The pooled analysis, with data from the concurrent BERNIE study, undertaken to overcome potential selection bias further confirmed the results presented for MTS 2008.

Both EFS and OS in the MTS 2008 study and the BERNIE study seem to be better than previously reported in a pooled analysis of data from 788 patients included in 9 European and North American studies between 1984 and 2000 (Table 3).^3^ The authors reported 3-year EFS of 27% (95% CI, 24 to 30) and 3-year OS of 34% (95% CI, 31 to 38), compared with 36% (95% CI, 30 to 41) and 49% (95% CI, 44 to 55), respectively, from the pooled analyses reported here. The results were similar to those achieved with the ARST0431 study (3-year EFS 38% [95% CI, 29 to 48] and 3-year OS 56% [95% CI, 46 to 66]).^2^ MTS 2008, BERNIE, and ARST0431 all introduced important changes to the treatment regimen, in particular the introduction of a years' maintenance treatment for both E*p*SSG studies and a dose-intensified, interval compressed regimen in ARST0431.

The concept of maintenance was suggested as a metronomic approach to kill residual tumor cells resistant to drugs given in prior standard chemotherapy.^13^ There is now convincing evidence for this approach in localized RMS. Recently, the E*p*SSG RMS 2005 trial showed that 24 weeks of maintenance with vinorelbine and low-dose cyclophosphamide improved OS in high-risk localized RMS.^8^

The possible contribution of prolonged vinorelbine and cyclophosphamide to the outcomes in the MTS 2008 cohort reported here is uncertain. For those patients who experienced an EFS event, the median time from diagnosis to event was 11.6 months (range, 0.2-63.8 months) for MTS 2008 patients, and 60/181 patients had an event during maintenance therapy, suggesting early failure. This remains a significant issue, and enhanced induction strategies are needed for such patients. By contrast, in patients with high-risk localized RMS, the median time from random assignment to relapse was delayed from 6.9 months (interquartile range, 3.0-16.1 months) to 10.1 months (interquartile range, 6.9-15.4 months) by the addition of 24 weeks of maintenance vinorelbine and cyclophosphamide, with the majority of events in both groups taking place after the 24-week window for maintenance treatment.^2^ The steep decrease in the survival curves presented in this report underlines the problem that the current systemic treatment approach of induction plus maintenance chemotherapy is insufficient to control the disease in many patients with metastatic RMS, especially in patients with adverse prognostic factors (ie, with 3-4 ORFs).

Anthracyclines were part of previous European regimens for metastatic disease^7,8^ and localized disease.^1^ The dose-intense addition of doxorubicin to the IVA backbone did not improve outcome in RMS 2005 in patients with high-risk localized RMS.^1^ Anthracyclines were also incorporated in two COG studies for patients with metastatic RMS (ARST0431 and ARST08P1; Table 3).^4,14^ Although ARST08P1 contained the same dose-dense chemotherapy backbone, including doxorubicin, and prolonged duration as the (historical comparison) ARST0431 study, outcome was inferior and failed to reveal the same trend in outcome improvement observed with both ARST0431 and the current study. This difference may be explained by the adjusted eligibility criteria in ARST08P1, where patients with favorable characteristics (age < 10 years and embryonal histology) were not eligible until safety was established, with a resulting different distribution of patient characteristics and ORFs. Nevertheless, these outcomes underline the limitations of comparisons between sequential studies. Although doxorubicin is an active drug in newly diagnosed metastatic RMS,^15^ the value of adding doxorubicin to a dose-dense chemotherapy backbone remains debatable.

Because of the design of the studies, the exact contributions of dose-intense doxorubicin and maintenance chemotherapy remain uncertain, and alternative explanations for the moderate survival improvement (compared with the historical cohort described by Oberlin et al) should be considered. First, more rigorous application of local treatment (ie, surgery and radiotherapy) may have improved outcome.^16-18^ Second, the systematic implementation of more effective second-line treatment^19^ may have prolonged postrecurrence survival. Finally, over the past decades, staging techniques and risk stratification have further evolved in addition to better supportive care treatments.

Previous studies in metastatic RMS categorized patients into poor and better outcome groups by comparing patients with 0-1 ORFs with patients having ≥ 2 ORFs.^3,4^ In the analyses presented in this study, this difference remained, but the EFS curves by ORFs were distributed differently from the curves presented previously: patients with 2 ORFs seemed to do better and group with the EFS curves for patients with 0 or 1 ORF. Although outside the scope of this study, it could be hypothesized that development in staging procedures, such as the increased use of ^18^F-labeled fluorodeoxyglucose positron emission tomography-computed tomography, may have resulted in the detection of more metastatic sites, moving patients with extensive disease, who previously may have been underdiagnosed and grouped as having 2 ORFs, to the group of patients with 3 or 4 ORFs. This may have resulted in improved survival figures for patients with 2 ORFs in the current pooled studies.

Unexpectedly, 3-year OS was lower in the MTS 2008 study compared with the BERNIE study. Any effect of bevacizumab can be discounted as it improved neither EFS^9^ nor OS (Fig 2) for patients with RMS within the BERNIE study. The BERNIE study was open in selected sites only, whereas the MTS 2008 study was open in all E*p*SSG centers. There were some minor differences between the studies in eligibility criteria (Data Supplement) and the method of response assessment (volumetric assessments in MTS 2008, RECIST 1.0 in BERNIE). After adjustment for known confounders, such as different age categories and eligibility of patients with CNS metastases, the survival difference became statistically nonsignificant. Comparisons between different studies should be made cautiously; other potential confounding factors in this analysis may be variability in eligibility criteria, data collection, or the limited number of patients in the BERNIE cohort (especially after 3 years of follow-up).

In conclusion, outcome for patients with high Oberlin scores remains very poor, and new approaches are needed for this patient group. In the recently opened E*p*SSG Frontline and Relapse Rhabdomyosarcoma study (EudraCT: 2018-000515-24), a phase Ib dose-finding study in patients with metastatic RMS will set the recommended phase II dose of irinotecan for the dose-intense combination of IVA in week 1 with irinotecan in week 2 (I_R_IVA).^10^ Patients with metastatic disease will then be randomly assigned to receive either IVADo or I_R_IVA at recommended phase II dose. In a second randomized question, 12 months of maintenance chemotherapy will be compared with 24 months maintenance therapy. Furthermore, there will be three random assignments on radiotherapy-related questions. Finally, the relapse part of the study will introduce targeted agents in combination with backbone chemotherapy.

## Data Availability

The data used in this study will be made available via the International Soft Tissue Sarcoma Consortium (INSTRuCT; https://commons.cri.uchicago.edu/instruct). Data requests can be submitted to INSTRuCT.
